# The impact of CSF gusher on preservation of low frequency residual hearing following cochlear implantation

**DOI:** 10.12669/pjms.42.5.12688

**Published:** 2026-05

**Authors:** Ghulam Fatima, Rimsha Jabeen, Jawwad Ahmad, Muhammad Jahangeer Badar, Junaid Shahzad, Ghulam Saqulain

**Affiliations:** 1 Dr. Ghulam Fatima, MBBS. Post Graduate Trainee, Department of ENT & CDA Cochlear Implant Centre, Capital Hospital PGMI, Islamabad, Pakistan; 2 Ms. Rimsha Jabeen, BSc (Audiology). Audiologist, Department of ENT & CDA Cochlear Implant Centre, Capital Hospital PGMI, Islamabad, Pakistan; 3 Dr. Jawad Ahmad, FCPS. Cochlear Implant Surgeon, Department of ENT & CDA Cochlear Implant Centre, Capital Hospital PGMI, Islamabad, Pakistan; 4 Mr. Muhammad Jahangeer Badar, MPhil (Audiology). Audiologist, Department of ENT & CDA Cochlear Implant Centre, Capital Hospital PGMI, Islamabad, Pakistan; 5 Dr. Junaid Shahzad, FCPS. Senior Registrar, Department of ENT & CDA Cochlear Implant Centre, Capital Hospital PGMI, Islamabad, Pakistan; 6 Dr. Ghulam Saqulain, FCPS. Consultant ENT Surgeon, Riphah Intenational Hospital, Islamabad, Pakistan. Ex. Head of Department, Department of ENT & CDA Cochlear Implant Centre, Capital Hospital PGMI, Islamabad, Pakistan

**Keywords:** Cochlear Implantation, CSF gusher, Hearing Impaired, Residual hearing

## Abstract

**Objective:**

To investigate and compare low-frequency residual hearing preservation following cochlear implantation in patients with or without an intraoperative cerebrospinal fluid (CSF) gusher.

**Methodology:**

This descriptive comparative cohort study was conducted at CDA Cochlear Implant Centre, Department of Otorhinolaryngology, Capital Hospital Islamabad, from 1^st^ April 2024 to 31^st^ March 2025. The study Utilized a sample of 70 cochlear implant recipients categorized into two groups based on the presence (n=12) or absence (n=58) of intraoperative CSF gusher. All patients underwent cochlear implantation with a standard electrode array (Manufactured by Cochlear, MED-EL) using soft surgical techniques and perioperative steroid administration and full insertion was achieved to maintain procedural uniformity and reproducibility. Low-frequency hearing thresholds were measured preoperatively and at 1, 3 and 6 months postoperatively to evaluate hearing preservation outcomes. SPSS Version 23 was used for data analysis.

**Results:**

The mean hearing thresholds (dB HL) for the Non-Gusher groups across all the four time points consistently exhibited lower thresholds. At the last follow-up, statistically significant (p=0.02) differences were observed between the groups with complete hearing preservation achieved in 33.3% of the CSF Gusher group compared to 44.8% in the Non-Gusher group, while significantly (p=0.05) more hearing loss in the CSF Gusher group. The mean change in threshold was significantly higher (p=0.05) in the Gusher Group.

**Conclusion:**

The presence of intraoperative CSF gusher is associated with a statistically significant decline in low-frequency residual hearing following cochlear implantation.

## INTRODUCTION

Cochlear implant (CI) is a surgically implanted device that bypasses non-functional sensory cells within the cochlea and directly stimulates the auditory nerve, effectively restoring hearing in individuals with impaired cochlear function.^1^ It has revolutionized the treatment of severe to profound sensorineural hearing loss, providing life-changing auditory benefits for both pediatric and adult populations. Since the first successful CI surgery in 1957, the field has evolved significantly, with over 700,000 cochlear implants now implanted globally, a figure that continues to rise annually, CI candidacy was limited to individuals with bilateral profound hearing loss. However, over the past two decades, the criteria for eligibility have expanded, allowing patients with residual low-frequency hearing to also benefit from CI.^2^

This expansion has been facilitated by advancements in surgical techniques, such as “soft surgery” methods, which minimize cochlear trauma during electrode insertion. Introduced by Lehnhardt in 1993, these techniques emphasize atraumatic electrode placement, the use of devices designed to minimize endocochlear damage, and perioperative administration of lubricants and protective agents.^3^ Literature has demonstrated a marked improvement in surgical outcomes, particularly in terms of preserving residual hearing and enhancing post-operative quality of life for patients.^4^ While CI is widely regarded as a safe and effective procedure, with an overall complication rate of approximately 12.5%, it is not without challenges. One of the most significant intraoperative complications is the occurrence of cerebrospinal fluid (CSF) gushers, which affect approximately 1-5% of CI surgeries.^5^

CSF gusher is defined as the initial pulsatile egress of profuse and clear fluid upon making an opening into inner ear, which can last for a few minutes before abating.^6^ This condition is distinct from the perilymphatic gushers observed in earlier stapedectomy surgeries, as the fluid involved in CI is CSF rather than perilymph due to it being a few microliters.^7^ CSF gusher is associated with different inner ear malformations such as incomplete partition anomalies, common cavity, cochlear hypoplasia and vestibular aqueduct syndrome.^8^ Residual hearing preservation is a critical goal in CI surgery, as it significantly enhances speech perception, musical tone recognition and overall auditory functions.^6^ Moreover, the ability to preserve low-frequency hearing is an important prognostic indicator for successful CI outcomes. Effective preservation relies on the precise placement of the CI electrode within the scala tympani, ensuring optimal nerve stimulation. However, complications such as CSF gushers may compromise this delicate process, as rapid fluctuations in intracochlear pressure during surgery can lead to irreversible damage to cochlear structures responsible for low-frequency hearing.^6^ Existing studies have demonstrated degrees of residual hearing preservation in patients without intraoperative complications.

A study by Mamelle et al. (2020) reported a 93% preservation rate of low-frequency residual hearing immediately after surgery, although this figure decreased over time.^9^ In contrast, patients who experience CSF may face a significant reduction in residual hearing, both immediately and in the long term. This is potentially due to rapid changes in intracochlear pressure during the manipulation of the round window membrane, which could disrupt cochlear integrity.^6^ Given the rising prevalence of sensorineural hearing loss and the growing inclusion of cochlear implantation in public health programs worldwide, it is essential to better understand the impact of intraoperative variables like CSF gushers on hearing outcomes.^10^ Therefore, current study was conducted to investigate and compare low-frequency residual hearing preservation following cochlear implantation in patients with or without an intraoperative cerebrospinal fluid (CSF) gusher. The study is of significant importance since it will provide critical insights into the relationship between CSF gushers and the preservation of low-frequency residual hearing, which could help refine preoperative counseling strategies and inform broader CI candidacy criteria, particularly for patients with varying degrees of residual hearing.

## METHODOLOGY

This descriptive comparative cohort study utilizing non-probability consecutive sampling was conducted at the CDA Cochlear implant Centre, Department of ENT, Capital Hospital Islamabad, over a period of one year from 1^st^ April 2024 to 31^st^ March 2025. The cohort included N=70 cochlear implant recipients of any age and gender who exhibited preoperative residual hearing at low frequencies of 250,500 and 1000 Hz. Those with any other disability or no preoperative residual hearing were excluded from the study.

The sample was categorized into two groups based on the presence (n=12) or absence (n=58) of intraoperative CSF gusher. The residual hearing before surgery and its preservation after cochlear implantation at low frequencies (250,500,1kHz) were defined as measurable unaided air conduction pure-tone thresholds ≤120db.^11^ The gusher group, included patients who experienced intraoperative CSF gusher. For comparison, the Non-gusher included patients with similar hearing pattern but with normal intraoperative findings.

### Ethical approval:

Ethical approval was obtained from the ethical review board of Capital Hospital, Islamabad vide registration # IRB- 27- 15-09-23 dated September 15, 2023. Informed consent was taken from all patients and parents of minors. Tools utilized for data collection included a basic demographic sheet and Pure Tone Audiometer (model: Maico MA 42).

All patients were evaluated by a multidisciplinary cochlear implant team comprising of otorhinolaryngologist, audiologists, speech therapists and pediatricians before enrolment in the CI program. Unaided air conduction pure tone hearing thresholds at 250,500 and 1kHz were measured preoperatively and postoperatively at one, three and six months. The frequency of residual hearing preservation was calculated using equation proposed by the HEARING GROUP. All patients underwent CT and MRI of the temporal bone preoperatively. Inner ear malformation were classified according to Sennaroglu classification.^12^ All cochlear implanation procedures were performed by the same surgeon using a standard round window approach. The basic elements of soft surgery were followed, ensuring slow insertion of electrodes via standard posterior tympanotomy approach, perioperative use of protective drugs and correct diagnosis of intraoperative CSF gusher. Standard electrode array used in all cases with full insertion achieved.

### Statistical analysis:

Data collected was entered and analyzed using SPSS 23.0. Descriptive statistics was calculated for both qualitative and quantitative variables. For qualitative variables (gender, preservation of low frequency residual hearing, CSF gusher and non-gusher) frequencies and percentages was calculated. For quantitative variables mean and standard deviation (SD) was calculated. Both qualitative and quantitative variables was presented through tables or graphs. Effect modifiers (age, gender) was controlled through stratification. Post-stratification chi-square was applied. Frequency of residual hearing preservation was compared among CSF gusher and non-gusher using chi-square test. P ≤ 0.05 was considered significant.

## RESULTS

This study included 12 (17.1%) children in the CSF Gusher group and 58(82.9%) in the Non-Gusher group with no significant difference between groups for male (p=0.08) and female(p=0.09) gender and age (p=0.10) (Table-I). Type of deformity differed (p=005), with a higher prevalence of IP-II variant and LVAS.

**Table-I:** Demographic and clinical characteristics of the sample population (n=70)

Variable	Group	CSF Gusher (n=12)	Non-Gusher (n=58)	P Value
Gender	Male	9 (75%)	42 (72.4%)	0.08
	Female	3 (25%)	16 (27.6%)	0.09
Age (years) Mean ± SD, Range		5.1 ± 8.2	4.3 ± 8.7	0.10
Type of Deformity				0.05
	IP-II Variant	5 (41.7%)	6 (10.3%)	0.08
	LVAS	4 (33.3%)	8 (13.8%)	0.05
	Cochlear Hypoplasia	1(8.3%)	0(0%)	0.10
	No Deformity	3 (25%)	44 (75.9%)	0.00
	Duration of Hearing Loss (years)	4.9 ± 2.1	4.1 ± 2.5	0.02

**Fig.1:**
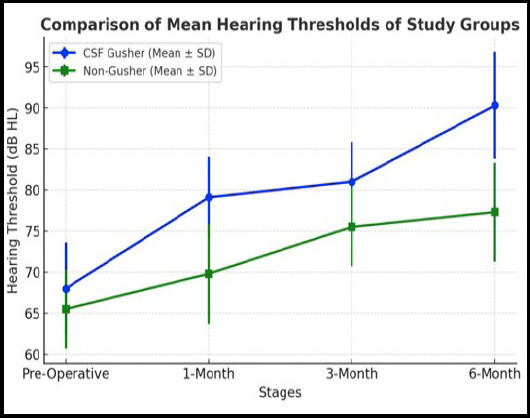
Comparison of Mean hearing thresholds of study groups pre-operatively and at three follow-ups.

The mean hearing thresholds (dB HL) for the Non-Gusher groups across all the four time points ie., pre-operative, 1-month follow-up, 3-month follow-up, and 6-month follow-up,consistently exhibited lower thresholds, indicating better hearing preservation compared to the CSF Gusher group. The error bars indicate greater variability in thresholds for the CSF Gusher group, reflecting increased fluctuations in hearing outcomes over time (Fig.1).

At the last follow-up, statistically significant (p=0.02) differences were observed between the CSF Gusher and Non-Gusher groups regarding hearing preservation (Table-II). Complete hearing preservation was achieved in 33.3% of the CSF Gusher group compared to 44.8% in the Non-Gusher group, while significantly (p=0.05) more hearing loss was reported in the CSF Gusher group. Mean pre-operative thresholds & mean post-operative threshold difference was not significant i.e., p=0.65 and p=0.70 respectively. However the mean change in threshold was significantly higher (p=0.05) in the Gusher Group.

**Table-II:** Comparison of primary and secondary outcomes among study groups at last followup (n=70)

Outcome	Group	CSF Gusher (n=12)	Non-Gusher (n=58)	Total (n=70)	p-Value
Hearing Preservation	Minimal Preservation (%)	2 (16.7%)	5 (8.6%)	7 (10%)	0.02
	Partial Preservation (%)	3 (25%)	15 (25.9%)	18 (25.7%)	0.06
	Complete Preservation (%)	4 (33.3%)	26 (44.8%)	30 (42.9%)	0.08
Loss of Hearing (%)		3 (25%)	12 (20.7%)	15 (21.4%)	0.05
Thresholds (dB HL)	Mean Pre-Operative Threshold	68.0 ± 5.6	65.5 ± 4.8	66.07± 5.1	0.65
	Mean Post-Operative Threshold	90.3 ± 6.5	77.3 ± 6.0	79.67± 6.4	0.70
Mean Change in Threshold		22.3	11.8	―	0.05

The post-stratification analysis revealed significant difference (p<0.05) in residual hearing preservation among various subgroups (Table-III). Females demonstrated a significantly (p=0.04) higher frequency of hearing preservation compared to males, with 94.7% females achieving preservation. Age also significantly (p=0.03), influenced hearing preservation, with children aged ≤9 years showing a preservation rate of 73.3% compared to 80% in those aged >10 years (p=0.03) (Table-III).

**Table-III:** Post-stratification comparison of frequency of residual hearing preservation among study groups

Variable	Subgroup	Preserved	Not Preserved	p-value
Gender	Male (n=51)	37/51 (72.5%)	14/51 (27.5%)	0.04
	Female (n=19)	18/19 (94.7%)	1/19 (5.3%)	0.04
Age	≤5 years (n=51)	43/51 (84.3%)	8/51 (15.6%)	0.03
	>5 years (n=19)	12/19 (63.15%)	7/19 (36.8%)	0.03
Type of Deformity	IP-II Variant	5/11 (45.5%)	6/11 (54.5%)	0.06
	LVAS	3/11 (27.3%)	8/11 (72.7%)	0.05
	Cochlear Hypoplasia	1/1 (100%)	0/1 (0%)	0.08
	No Deformity	47/47 (100%)	0/47 (0%)	0.07

The type of deformity played a critical role, with patients having no deformity exhibiting a 100% preservation rate, whereas those with IP-II Variant or LVAS deformities had much lower preservation rates of 45.5% and 27.3%, respectively (p=0.05). Patients with cochlear hypoplasia had a 100% preservation rate, though this was based on a single case.

## DISCUSSION

Our study aimed to assess the impact of intraoperative CSF gusher on the preservation of low frequency residual hearing following cochlear implantation, comparing outcomes between patients with and without a gusher. To the best of our knowledge, this represents the first study of its kind conducted in this country, contributing region specific insights to the existing body of international literature.

Among the 70 patients included, 55 patients (78.6%) maintained low-frequency residual hearing after surgery. However, those in the gusher group demonstrated a more substantial decline in residual hearing compared to non-gusher during the postoperative period. Despite this observed difference, cochlear implantation provided significant auditory benefits across all participants.

The etiology of CSF gusher in our sample is likely linked to inner ear anomalies such as modiolus deficiency, isolated enlarged vestibular aqueduct and cochlear hypoplasia.^12^ Although the cochlear aqueduct has been proposed as a potential route for CSF leakage, imaging in our cases did not found any abnormalities in its size or configuration.^13^

CSF gusher presents surgical challenges and may also predispose to adverse outcomes such as increases the risk of meningitis and suboptimal hearing results.^12^,^14^ Our study adds to the literature by exploring the influence of CSF gusher on residual hearing.

Other than the presence of intraoperative CSF gusher, both groups were similar. Both groups had patients with and without inner ear anomalies. Our findings suggest that CSF gusher has significant impact on residual hearing in the postoperative period.

The results of the present study align with those reported in previous international literature. Gautschi Mills K et al. reported a 92% success rate in preserving residual hearing, with approximately half of the participants demonstrating complete preservation across all frequencies postoperatively.^4^ Similarly, Balkany TJ et al. observed preservation of pure tone hearing in 89% of cases , with 32% of patients achieving complete preservation and 57% showing partial preservation at low frequencies of 250, 500 and 1000 Hz.^15^ Additionally, Di Nardo W et al. reported a 78% rate of low frequency hearing preservation in their study population.^16^ our study demonstrated a comparable overall preservation rate of 78.6% for low-frequency hearing, thereby reinforcing these international studies.

Bruschke S et al. observed that postoperative residual hearing decreased compared to preoperative levels; however, the hearing thresholds remained stable within the first year following CI.^17^ In contrast, our study found a more significant and immediate postoperative decline in residual hearing, particularly pronounced in the gusher group. This hearing loss was evident immediately after surgery and persisted over time. This is in accordance with the study carried out by Crohn W et al. who proposed that a sudden and rapid change in intracochlear pressure during gusher cause damage to cochlear hair cells, resulting in loss of residual hearing.^6^

The preservation of residual hearing through atraumatic and soft surgical techniques is well supported in literature.^18^-^20^ Verhaegen et al. reported mean corrected threshold differences of 25 dB at 250 Hz and 20 dB at 500 Hz in the classic implantation group, while the soft surgery group showed significantly smaller differences of 10 dB and 7.5 dB, respectively.^19^ Similarly, Fraysse B et al. found that more than 50% of patients retained measurable postoperative hearing threshold levels following soft surgery protocols.^20^ Furthermore, Gstoettner W et al. reported successful hearing preservation in 85.7% patients using these techniques.^19^ Derinsu U et al achieved preservation of low frequency residual hearing in 87% of patients with complete hearing preservation at all frequencies was accomplished in 35.48%.^21^ These findings are consistent with our results and further demonstrate the positive impact of soft surgical techniques on the preservation of residual hearing.

In the gusher group, residual hearing not only suffered an initial decline but also showed progressive deterioration over time. The mechanism for this continued loss is not entirely clear; however, it may be attributed to persistent alterations in intracochlear pressure along with postcochlear implantation inflammatory responses within the cochlea.^22^ It is also possible that the pharmacologic efficacy of topical steroid is reduced due to dilution or washout, particularly in the presence of excessive fluid flow within the cochlea.^6^ Despite the harmful effects of gusher on residual hearing, all patients received significant hearing benefit from cochlear implant.

Our study suggests that preservation of residual hearing is feasible following cochlear implantation particularly in cases without intraoperative complication i.e CSF gusher. As cochlear implant technology continues to evolve, this finding holds significant promise for optimizing hearing preservation and improving audiological outcomes, offering new possibilities for enhanced patient care.

### Limitations & Strengths:

This study provides valuable data on predictors of hearing preservation in cochlear implantation, particularly within a regional context where such evidence is limited. A key strength is the procedural consistency, as all surgeries were performed by a single experienced surgeon using a standardized soft surgery technique, which minimizes variability and strengthens the reliability of the findings. Morever, the use of the Sennaroglu radiological classification allows precise assessment of inner ear anomalies, strengthening the correlation between anatomy and hearing outcomes. Overall, the study offers clinically relevant insights that can guide surgical decision-making in complex cases.

Despite its clinical relevance, this study has few limitations. First, the CSF gusher subgroup (n=12) was small, limiting the power of subgroup comparisons. Second, as a single-center study, the findings may not fully reflect broader, multicenter demographics. Third, the follow-up period was limited to 6 months, While this duration was sufficient to observe immediate and short-term post-operative declines, most standard cochlear implant hearing preservation studies track outcomes up to 12 or 24 months to assess long-term stability. Finally, a multivariate analysis was not performed, so potential confounding factors—such as patient age, duration of hearing loss, electrode type, and specific inner ear malformations—could not be independently evaluated. Future multicenter studies with larger cohorts and extended follow-up are needed to address these limitations and validate these findings.

## CONCLUSIONS

The presence of a CSF gusher during cochlear implantation has a significant impact on the low frequency residual hearing. The study identified statistically significant differences between the CSF gusher group and the non-gusher group in terms of preservation rates or changes in low frequency hearing thresholds.

### Authors Contribution:

**SS:** Collection of data & statistical analysis.

**NM:** Critical revision of the article and supervision.

**SY:** Conceived and designed methodology.

**GS:** Did the writing of the manuscript and was responsible for the integrity of the research.

All authors have read and approved the final version of the manuscript.

## References

[1] Jwair S, Versnel H, Stokroos RJ, Thomeer HGXM (2022). The effect of the surgical approach and cochlear implant electrode on the structural integrity of the cochlea in human temporal bones. Sci Rep.

[2] Deep NL, Dowling EM, Jethanamest D, Carlson ML (2019). Cochlear Implantation: An Overview. J Neurol Surg B Skull Base.

[3] Lin CC, Chiu T, Chiou HP, Chang CM, Hsu CJ, Wu HP (2021). Residual hearing preservation for cochlear implantation surgery. Tzu Chi Med J.

[4] Gautschi-Mills K, Khoza-Shangase K, Pillay D (2019). Preservation of residual hearing after cochlear implant surgery: an exploration of residual hearing function in a group of recipients at cochlear implant units. Braz J Otorhinolaryngol.

[5] Hashemi SB, Bozorgi H, Kazemi T, Babaei A (2020). Cerebrospinal fluid gusher in cochlear implant and its associated factors. Acta Otolaryngol.

[6] Crohan W, Krishnaswamy J, Rajan G (2018). The Effects of Gusher-Related Intracochlear Pressure Changes on Hearing Preservation in Cochlear Implantation: A Comparative Series. Audiol Neurotol.

[7] Chauhan VM, Vishwakarma R (2019). CSF Gusher and Its Management in Cochlear Implant Patient with Enlarged Vestibular Aqueduct. Indian J Otolaryngol Head Neck Surg.

[8] Dalgic A, Atsal G, Ceylan ME, Aydın E, Adıbelli ZH, Edizer DT (2022). Cerebrospinal fluid gusher in cochlear implantation and its association with inner-ear malformations. J Int Adv Otol.

[9] Mamelle E, Granger B, Sterkers O, Lahlou G, Ferrary E, Nguyen Y, Mosnier I (2020). Long-term residual hearing in cochlear implanted adult patients who were candidates for electroacoustic stimulation. Eur Arch Otorhinolaryngol.

[10] Ahmed J, Saqulain G, Khan MI, Kausar M (2021). Complications of cochlear implant surgery: A public implant centre experience. Pak J Med Sci.

[11] Stuermer KJ, Kluenter HD, Lang-Roth R, Schwarz D, Hüttenbrink KB, Anagiotos A (2019). Preservation of vestibular function and residual hearing after round window cochlear implantation. Otol Neurotol.

[12] Sennaroğlu L, Bajin MD (2017). Classification and current management of inner ear malformations. Balkan Med J.

[13] Bianchin G, Polizzi V, Formigoni P, Russo C, Tribi L (2016). Cerebrospinal fluid leak in cochlear implantation: enlarged cochlear versus enlarged vestibular aqueduct (common cavity excluded). Int J Otolaryngol.

[14] Hazazi M, Almashharawi E, Alamry S, Alkusayer MM, Altimyat A, Alsalamah Y (2024). Retrospective analysis of cerebrospinal gushers in cochlear implant surgery: incidence, risk factors, and outcomes—a systematic review and meta-analysis. Ear Nose Throat J.

[15] Balkany TJ, Connell SS, Hodges AV, Payne SL, Telischi FF, Eshraghi AA (2006). Conservation of residual acoustic hearing after cochlear implantation. Otol Neurotol.

[16] Di Nardo W, Cantore I, Melillo P, Cianfrone F, Scorpecci A, Paludetti G (2007). Residual hearing in cochlear implant patients. Eur Arch Otorhinolaryngol.

[17] Bruschke S, Baumann U, Stöver T (2023). Residual low-frequency hearing after early device activation in cochlear implantation. Eur Arch Otorhinolaryngol.

[18] Nguyen S, Cloutier F, Philippon D, Côté M, Bussières R, Backous DD (2016). Outcomes review of modern hearing preservation technique in cochlear implant. Auris Nasus Larynx.

[19] Verhaegen VJ, Snik AF, Beynon AJ, Leeuw RA, Mylanus EA (2012). Preservation of low frequency residual hearing after cochlear implantation. Is soft surgery effective?Otol Neurotol.

[20] Fraysse B, Macías ÁR, Sterkers O, Burdo S, Ramsden R, Deguine O (2006). Residual hearing conservation and electroacoustic stimulation with the Nucleus 24 Contour Advance cochlear implant. Otol Neurotol.

[21] Derinsu U, Serin GM, Akdas F, Batman Ç (2011). Cochlear implantation: is hearing preservation necessary in severe to profound hearing loss?. J Craniofac Surg.

[22] Jia H, François F, Bourien J, Eybalin M, Lloyd RV, Van De Water TR (2016). Prevention of trauma-induced cochlear fibrosis using intracochlear application of antiinflammatory and antiproliferative drugs. Neuroscience.

